# A Denoising Method Using Deep Image Prior to Human-Target Detection Using MIMO FMCW Radar

**DOI:** 10.3390/s22239401

**Published:** 2022-12-02

**Authors:** Koji Endo, Kohei Yamamoto, Tomoaki Ohtsuki

**Affiliations:** 1 Graduate School of Science and Technology, Keio University, Yokohama 223-8522, Japan; 2 Department of Information and Computer Science, Faculty of Science and Technology, Keio University, Yokohama 223-8522, Japan

**Keywords:** radar, denoising, deep image prior

## Abstract

A Multiple-Input Multiple-Output (MIMO) Frequency-Modulated Continuous Wave (FMCW) radar can provide a range-angle map that expresses the signal power against each range and angle. It is possible to estimate object locations by detecting the signal power that exceeds a threshold using an algorithm, such as Constant False Alarm Rate (CFAR). However, noise and multipath components often exist over the range-angle map, which could produce false alarms for an undesired location depending on the threshold setting. In other words, the threshold setting is sensitive in noisy range-angle maps. Therefore, if the noise is reduced, the threshold can be easily set to reduce the number of false alarms. In this paper, we propose a method that improves the CFAR threshold tolerance by denoising a range-angle map using Deep Image Prior (DIP). DIP is an unsupervised deep-learning technique that enables image denoising. In the proposed method, DIP is applied to the range-angle map calculated by the Curve-Length (CL) method, and then the object location is detected over the denoised range-angle map based on Cell-Averaging CFAR (CA-CFAR), which is a typical threshold setting algorithm. Through the experiments to estimate human locations in indoor environments, we confirmed that the proposed method with DIP reduced the number of false alarms and estimated the human location accurately while improving the tolerance of the threshold setting, compared to the method without DIP.

## 1. Introduction

Radar-sensing technology has great potential for the development of various applications, such as object sensing via automotive radar. A Multiple-Input Multiple-Output (MIMO) radar equips array antennas and can estimate the Direction of Arrival (DOA) by analyzing the received signals of the MIMO radar. By adjusting the beam direction based on the estimated DOA, it could be possible to improve the Signal-to-Noise Ratio (SNR) of the desired signal. A MIMO Frequency-Modulated Continuous Wave (FMCW) radar is an FMCW radar with array antennas.

In addition to the DOA, the MIMO FMCW radar can estimate the range between a radar and an object, which means that this radar can estimate object locations. The object location information is essential for applications with the MIMO FMCW radar, e.g., object identification [1,2], activity recognition [3,4,5] and vital sign detection [6,7,8,9,10]. Thus, there is the need to estimate object locations with high accuracy [11,12,13,14,15,16,17,18].

To estimate an object’s location via the MIMO FMCW radar, a range-angle map that expresses the signal power against each range and angle is typically calculated. The location with the signal power that exceeds a threshold is detected over the range-angle map using a threshold setting algorithm, such as the Constant False Alarm Rate (CFAR) [19]. However, the object-detection accuracy could degrade depending on the threshold setting.

In particular, a location where a desired object does not exist could be detected incorrectly due to noise and multipath components. In other words, the threshold setting is sensitive in the noisy range-angle map. Therefore, if the noise is more reduced, the threshold can be set flexibly to reduce the number of false alarms. Although the method to reduce the effect of clutter noise using the Curve-Length (CL) method was previously investigated [20], false alarms of undesired locations can still happen even in this method.

Therefore, we consider reducing noise from range-angle maps by deep learning. In the field of vehicle-detection radar, a similar research has been conducted to detect target of applying the autoencoder in the range-angle map [21]. In [21], the autoencoder is used for range-angle maps to achieve high detection performance; however, prior training is required. When considering person detection that can be used anywhere, it is desirable to be able to detect targets without prior learning.

Here, note that “Tolerance” is defined as the range of the CFAR threshold that detects targets without false alarms. To realize location estimation with less sensitivity to the threshold setting to detect targets in this paper, we propose a method that improves the CFAR threshold tolerance by denoising a range-angle map using Deep Image Prior (DIP). DIP is one of the state-of-the-art unsupervised deep-learning techniques to remove noise components from an image.

Due to the advantages of the denoising ability without pre-training, DIP has been successfully applied in various fields, such as wireless communications [22]. To reduce the noise that causes a false alarm when applying DIP, it is important to decide how to best apply DIP. In MIMO FMCW radar, beamforming and range FFT are applied to the IQ signals obtained by the antennas to obtain IQ signals for range-angle maps. It is then necessary to detect targets based on the characteristics of the IQ signal at each range angle obtained.

We first applied DIP to IQ signals received at antennas in both simulations and actual measurements. Although we confirmed that the IQ signals with applied DIP improved the SNR of the angular spectrum by beamforming, the phase information was corrupted, resulting in multiple peak separations or accuracy degradation. Since the SNR of the beamforming angular spectrum is improved, DIP may be able to reduce the intensity of uncorrelated noise that is difficult to integrate while maintaining the intensity of highly correlated signals that are easy to integrate, such as signals emitted from one direction.

Based on the above, we considered applying DIP to signals that do not have phase information and are highly correlated at the target location. When the person is the target, the phase trajectory lengths produced by human motion are considered to be highly correlated, while other reflected waves are considered to be less correlated. Based on the above, we propose a method to remove noise by applying DIP after generating signals without phase information while maintaining the correlation of human signals.

In the proposed method, a range-angle map is calculated based on related work [20] from received signals of the MIMO FMCW radar. In this range-angle map, the clutter noise remains at a level that can cause false alarms. DIP is then applied to the range-angle map for noise reduction without pre-training. An object’s location is detected by using Cell-Averaging CFAR, which is a typical adaptive threshold setting algorithm. The application of DIP improves the threshold tolerance of CA-CFAR, which improves the detection and reduces false alarms.

To evaluate the location-estimation accuracy of our proposed method, we performed preliminary experiments to estimate human location with the MIMO FMCW radar in indoor environments [23]. Our previous studies have shown that DIP improves the detection performance, such as the Receiver Operating Characteristics (ROC) curves and CFAR threshold setting under limited conditions. We investigated the relationship between the location-estimation accuracy of our method and the threshold setting. The experimental results showed that the proposed method with DIP could reduce the number of false alarms and detect the human location while improving the tolerance of the threshold setting, compared to the method without DIP.

In summary, the contributions of our research are as follows.

- We clarified how we should apply DIP to improve the human-target-detection performance.
- We show that the proposed method improves the target-detection performance and simplifies the setup for detection by actual measurements.
- The proposed method is shown to be effective compared to other denoising methods that require learning.

The rest of this paper is organized as follows. In Section 2, we describe the principle of the MIMO FMCW radar. We then explain our proposed method to detect human targets in Section 3. In Section 4, we evaluate the performance of our method through experiments. Finally, we conclude this paper in Section 5.

## 2. Principle of MIMO FMCW Radar

In this section, we first explain the principle of the FMCW radar and then explain the MIMO FMCW radar. The FMCW radar sweeps the frequency of the transmitted microwaves linearly. Now, let $f_{c}$ and *B* be the minimum sweep frequency and the bandwidth of the chirp, respectively. Furthermore, let $T_{c}$ be the sweep duration. In the FMCW radar, microwaves are transmitted by the transmitting antenna, and the transmitted microwaves are reflected by objects. The reflected signal r(t) is then received by the receiving antenna.

(1) $$\begin{array}{c} \begin{array}{c} r\left(t\right)=A\{\cos\left(2\pi f_{c}\left(t-t_{d}\right)+\pi \frac{B}{T_{c}}{\left(t-t_{d}\right)}^{2}+\phi \left(t-t_{d}\right)\right)\}, \end{array} \end{array}$$

where *A* is the signal power and ϕ(t) is the phase noise. Furthermore, $t_{d}$ is given as $t_{d}=\frac{2R(t)}{c}$, where R(t) is the range between the FMCW radar and the subject, and *c* is the speed of the electromagnetic wave. The received signal is modulated into two signals with the phase difference of π/2, i.e., in-phase and quadrature signals. These two signals can be expressed as a complex signal y(t).

(2) $$\begin{array}{c} y\left(t\right)=Ae^{j(2\pi f_{b}t+\Phi \left(t\right)+\Delta \phi \left(t\right))}, \end{array}$$

where $f_{b}=2BR\left(t\right)/\left(cT_{c}\right)$, $\Phi \left(t\right)=2\pi f_{c}t_{d}+\pi Bt_{d}^{2}/T_{c}$, and $\Delta \phi \left(t\right)=\phi \left(t\right)-\phi \left(t-2R/c\right)$.

In the MIMO FMCW radar, where $N_{Tx}$ transmitting antennas and $N_{Rx}$ receiving antennas are linearly arranged, $N_{Tx}\times N_{Rx}$ virtual array antennas are constructed. When the reflected signal with the incident angle of θ is received at the *k* th antenna of *K* virtual array antennas, the received signal $y_{k}\left(t\right)$ is expressed as

(3) $$\begin{array}{c} y_{k}\left(t\right)=Ae^{j(2\pi f_{b}t+\Phi \left(t\right)+\Delta \phi \left(t\right)+\frac{2\pi }{\lambda }d_{k}\sin\theta )}, \end{array}$$

where λ is the wavelength of the carrier, and $d_{k}$ is the relative distance between the receiving antenna and the reference point. By calculating the beamforming weight $w_{k}$, the received signal for a specific beam direction can be obtained as

(4) $$\begin{array}{c} Y\left(t\right)=\sum_{k=1}^{K}y_{k}\left(t\right)w_{k}. \end{array}$$

## 3. Proposed Human-Target Detection from Radar Signals

**Figure 1:**
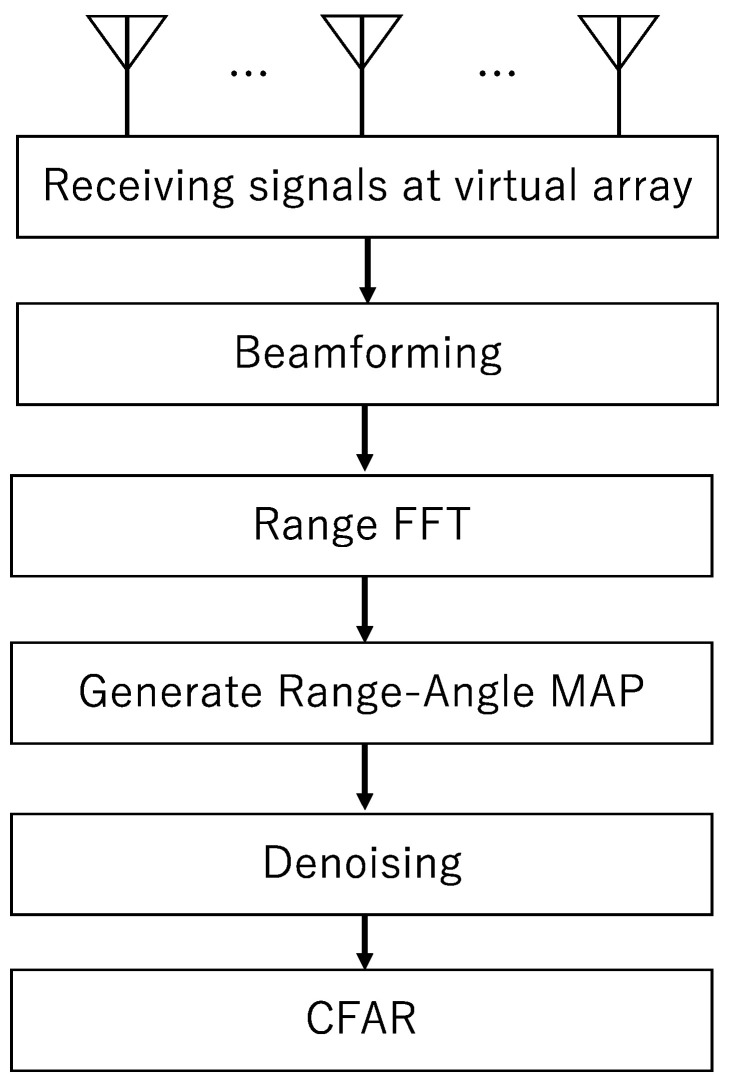
The flowchart of the proposed method.

In this section, we explain the algorithm of the proposed method for human-target detection from radar signals. Figure 1 shows the flowchart of our proposed method. Algorithm 1 shows the algorithm of our proposed method.
**Algorithm 1:** The algorithm of the proposed method.
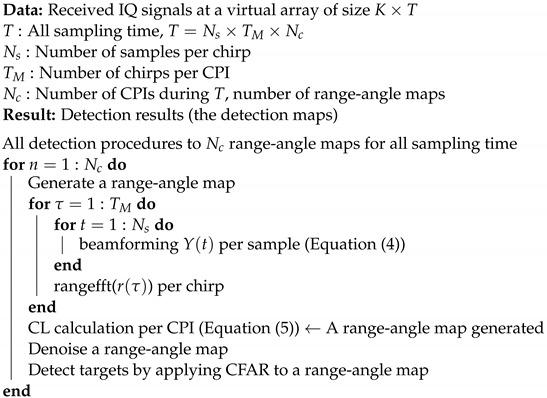


### 3.1. Range Angle MAP

In the proposed method, the range FFT is first applied to Y(t) for each beam direction. The range-angle map is then calculated based on the Curve-Length (CL) method [20]. The CL method calculates the length of the variability of IQ signals on the IQ plane within the Coherent Processing Interval (CPI) at every specified range and angle as

(5) $$\begin{array}{c} \begin{array}{c} CL=\sqrt{\sum_{\tau =2}^{T_{M}}\left({\left(I\left[\tau \right]-I\left[\tau -1\right]\right)}^{2}+{\left(Q\left[\tau \right]-Q\left[\tau -1\right]\right)}^{2}\right)}, \end{array} \end{array}$$

where $T_{M}$ is a CPI, and I[τ] and Q[τ] are the in-phase and quadrature signals on a specified range, angle and sampling time τ, respectively.

### 3.2. Human-Target Detection

Finally, the location estimation is performed over the denoised range-angle map based on Cell-Averaging (CA) CFAR. In CA-CFAR, target detection is performed using the following procedure. First, a cell under the test is selected over the range-angle map. A guard band around the cell under the test and a training band around a guard band are set. A threshold $V_{th}$ is determined as the following equation.

(6) $$\begin{array}{c} V_{th}=\mu \alpha , \end{array}$$

where μ is the average value of the signals in the training band that denotes the noise, and α is a specified factor. If the value under the test cell exceeds the threshold $V_{th}$, the cell is detected. After performing the above procedures for all the cells over the range-angle map, we can obtain a detection map where “1” means the detected cell, and “0” means the cell that is not detected. In these procedures, to detect targets and avoid false alarms, α must be set appropriately.

### 3.3. Effects of Noise

The range-angle map described in Section 3.1 can contain noise as well as targets. In this noisy environment, it is necessary to set α appropriately as described in the previous section in order to properly detect targets without false detection of noise using CFAR. If α is increased to reduce false alarms due to noise, the threshold $V_{th}$ will also increase, making target detection difficult and challenging. If α is made smaller to ensure reliable target detection, the threshold value $V_{th}$ also becomes smaller, resulting in false detection due to noise.

Therefore, in noisy environments, the possible range of α for target detection becomes narrower, and it becomes difficult and sensitive to drive the setting. If we can reduce the noise in the range-angle map, the possible range of α for target detection becomes wider, and we can set α more flexibly. Therefore, in this paper, we define “tolerance” as the possible range of α for target detection and aim to improve the target-detection performance by reducing noise from range-angle maps and widening the tolerance.

### 3.4. Denoising

According to the previous subsection, we need to denoise the range-angle map to reduce the effects of noise and multipath components. Here, we apply two types of denoising methods, Denoising Convolutional Neural Network (DnCNN) as a supervised denoising method and Deep Image Prior (DIP) as an unsupervised denoising method.

#### 3.4.1. Denoising Convolutional Neural Network (DnCNN)

DnCNN is an image denoising method that achieves denoising by removing the signal, estimating the noise and removing the estimated noise from the image. It is also used to improve the accuracy of spectrum estimation for noisy signals [24]. In this paper, DnCNN was implemented in MATLAB’s Toolbox as in [24]. MATLAB’s Deep Learning Toolbox offers two types of denoising: denoising using a pre-trained network and denoising by learning from images [25].

**Figure 2:**
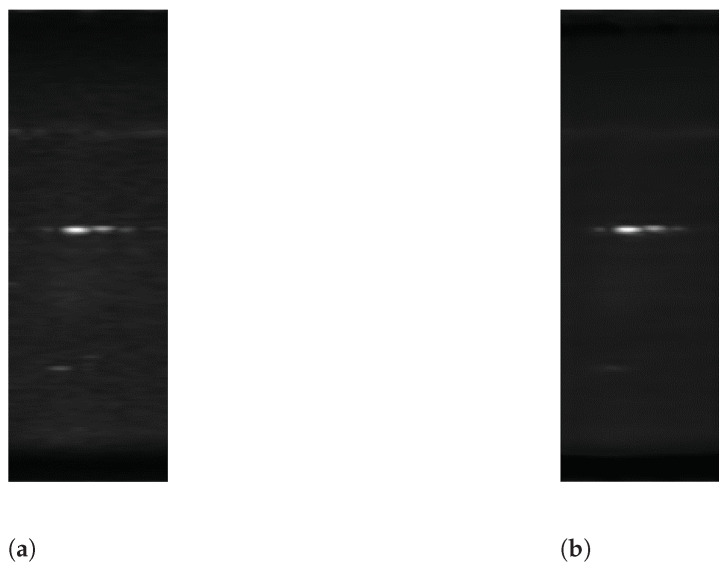
Noisy range-angle maps. (**a**) A noisy range-angle map. (**b**) A range-angle map denoised by a trained network.

Denoising using a pre-trained network is a method for removing Gaussian noise from images using a network optimized for removing Gaussian noise. The method of training the denoising network with an embedded layer allows the network to be trained to detect a wide range of Gaussian noise standard deviations. In this paper, we prepare the range-angle maps with targets as shown in Figure 2a and use these images to train the network. Next, the learned network is used to generate noise-reduced range-angle maps, such as Figure 2b. Hereafter, DnCNN used in this paper will be a network trained by experimental data unless explicitly stated as a pre-trained network.

#### 3.4.2. Deep Image Prior (DIP)

DIP is a method to obtain denoising, superresolution and inpainting effects on an image by using the structure of neural networks without prior training [26]. As a method for noise removal, we focus on the denoising effect of DIP [27]. DIP is a Deep Neural Network (DNN) model that realizes image processing, such as noise removal, without the use of supervised data. To extract a natural image with the noise removed from a target image, a random image is input to the DIP network, and the image is output. The parameters of the network are updated so that the loss function between the network output and the target image becomes smaller as follows.

(7) $$\begin{array}{c} \theta^{*}=\arg\min_{\theta }E\left(f_{\theta }\left(z\right);x_{0}\right), \end{array}$$

where θ is the parameter of a network, *z* is the input image, $x_{0}$ is the target image containing noise, *f* is the mapping function of the network, and $E\left(x;x_{0}\right)=||x-x_{0}{\left|\right|}^{2}$ is the loss function. By stopping training in the process of repeat image restoration, the network outputs an image with the noise removed from the target image. The transitions of the loss function by DIP for different images are shown in [27]. When comparing the noise image and the natural image, there is a difference in the number of epochs at which the value of the loss function decreases.

As shown in [26,27], the loss function decreases with a smaller number of epochs in the natural image. Thus, a natural image is restored faster than a random image in DIP. Therefore, by stopping training before the noise is restored, a noise-removed image is output. DIP is also applied to improve the accuracy of channel estimation by applying it to the heatmap of the received signals [22]. Since the range-angle map is a mixture of natural images consisting of signals reflected by the target and noise images consisting of multipath and noise components, it may be possible to remove the noise of the received signals by applying DIP to the range-angle map, similar to the problem of image restoration. In this paper, we apply the denoising method of DIP to images of range-angle maps.

## 4. Experimental Evaluation

To evaluate the performance of the proposed method, we conducted experiments to estimate human locations in indoor environments. In this section, we first explain the experimental setup and then present the experimental results.

### 4.1. Experimental Setup

**Figure 3:**
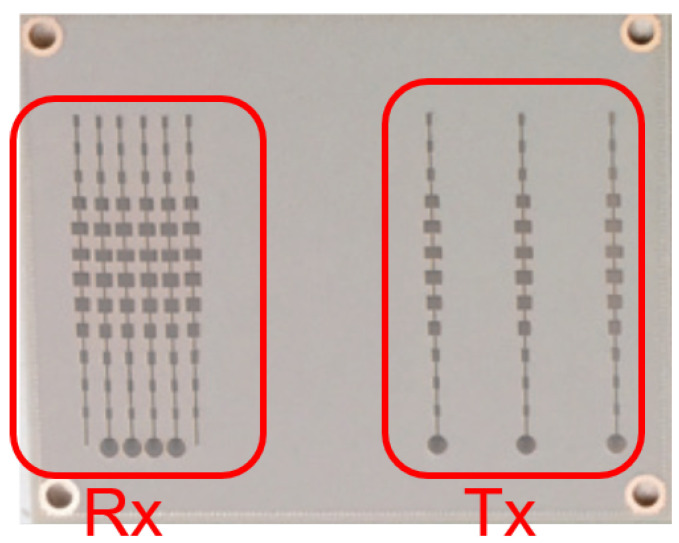
MIMO FMCW radar.

In the experiments, we conducted data recording using a 79 GHz MIMO FMCW radar with 12 virtual element antennas as shown in Figure 3. Table 1 lists the radar specifications. The MIMO FMCW radar consists of a linear array of three transmitting and four receiving antennas. The distance between two adjacent transmitting antennas is twice as long as the wavelengths. Furthermore, the distance between two adjacent receiving antennas is half of a wavelength.

By transmitting chirp signals from each transmitting antenna in order and receiving them at each receiving antenna, we acquired 12 modulated chirp signals. These signals are equivalent to the signals received by the 12 receiving linear array antennas. In the experiments, the MIMO FMCW radar was oriented horizontally so that the linear array antennas of the radar were arranged horizontally.

**Figure 4:**
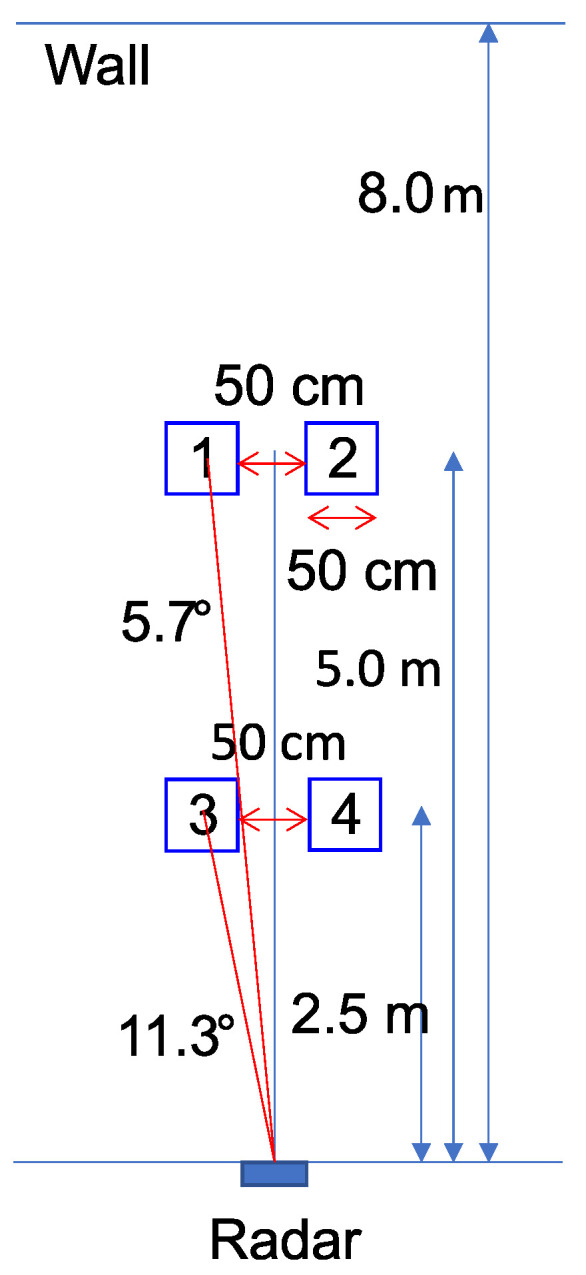
The experimental environment, where “1”, “2”, “3”, and “4” mean the locations people were seated.

**Figure 5:**
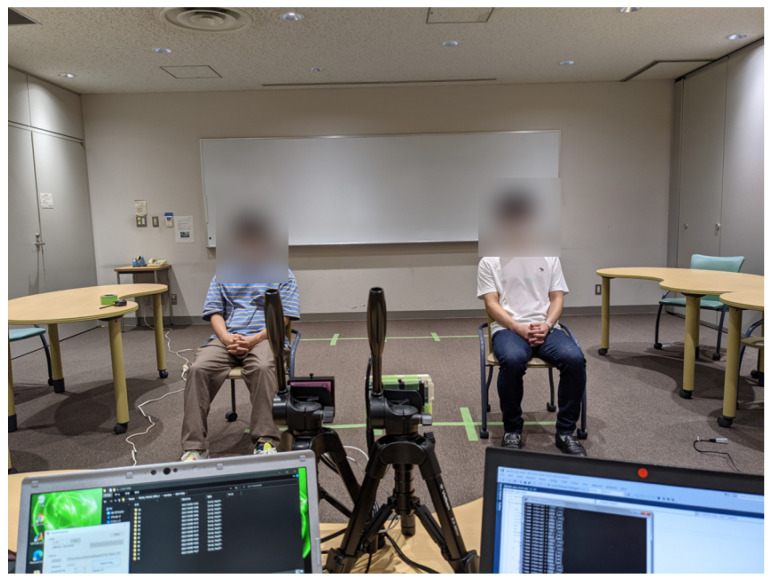
The experimental environment at location (3,4).

Figure 4 and Figure 5 show the experimental environment. In the experiments, two people were seated in two of the locations 1, 2, 3 and 4 as shown in Figure 4. MIMO FMCW radar can detect multiple targets simultaneously because it can obtain signals for each range and angle by generating range-angle maps. We chose the above location to simplify the evaluation of the target-detection performance in subsequent evaluations. The locations 1 and 2 were about 5.0 m away from the radar, while locations 3 and 4 were about 2.5 m away from the radar.

Figure 5 shows an example of the experimental environment where two subjects exists at locations 3 and 4. There was a wall about 8.0 m from the radar. The observation duration was 30 s, with an extra 30 s of measurement for training data for DnCNN. Furthermore, we performed the performance evaluation based on the CA-CFAR parameters as shown in Table 2. To implement the code of DnCNN and DIP, we refer to [25,28], respectively.

### 4.2. Results

In this section, we present the results with and without denoising based on the data measured with the parameters described in the previous section. DIP and DnCNN were applied for denoising. We demonstrate the advantages of the proposed method by processing the obtained data as follows:

1. Generate the range-angle maps by applying beamforming, range FFT and the CL method on the received signals.
2. Generate the detection maps by applying CFAR on the range-angle maps.
3. Calculate the detection rate, the false-alarm rate and the tolerance of the CFAR parameter.
4. Apply denoising to the range-angle maps processed in step 1 and then apply steps 2 and 3.
5. Evaluate the target-detection performance of the conventional method obtained in steps 1 through 3, and the proposed method obtained in step 4.

In step 1, denoising is not applied to preserve the phase of the IQ signal, and a signal that emphasizes the human body without phase information is generated. In step 2, target-detection processing is performed, and the results are used to check the target-detection performance and the appropriate range of the CFAR parameters in step 3. Using these values, the improvements by the proposed method are evaluated later. Step 4 is the proposed method, which applies DIP to the signals obtained in step 1 that are suitable for denoising by DIP. This step improves the target-detection performance and the appropriate range of CFAR parameters, which are evaluated below.

The evaluation in step 5 is presented in this section as follows. We first show examples of the effects of denoising on the range-angle maps and the detection maps. The range-angle maps with the proposed method intuitively confirm that the effect of noise was reduced, and the detection maps processed by CFAR with the proposed method show that false alarms were reduced. Next, we evaluate the target-detection performance after denoising. By evaluating the detection rate and false-alarm rate for varying CFAR thresholds, we show that the proposed method improved the target-detection performance. Then, we compare the tolerance of the CFAR parameter, which must be set a priori.

By evaluating the range of the CFAR parameters that can detect targets and not cause false alarms, we show that the proposed method extends the tolerance. In addition, we analyze the SNR of the range-angle maps for the following two points. By evaluating the SNR of the range-angle maps, we show that the SNR was improved by the proposed method. By applying denoising to the noise-added range-angle maps, we show that the proposed method improved the target-detection performance even in noisier environments.

#### 4.2.1. Comparison of Range-Angle Maps

**Figure 6:**
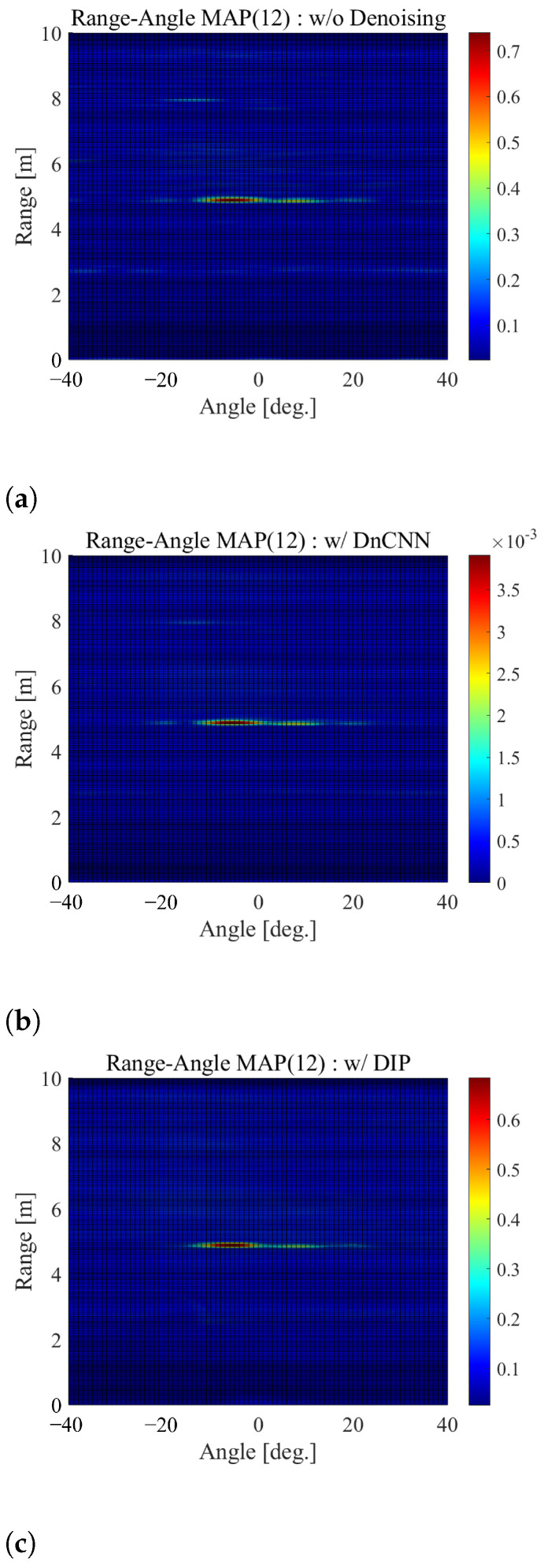
The comparison of range-angle maps (**a**) without denoising, (**b**) with DnCNN and (**c**) with DIP.

First, a comparison is made for the range-angle maps. In Figure 6a–c, we show examples of the range-angle maps without denoising, with DnCNN and with DIP, respectively. Here, note that two people exist at locations 1 and 2. Furthermore, the range-angle map is calculated using 1 sec I/Q data corresponding to 160 samples.

In Figure 6a, we can see that the cell values around 5.0 m are large, which is due to the two people. From this figure, it can be also seen that the noise components appear around 8.0 m, which could be detected based on CA-CFAR unintentionally. In contrast, in Figure 6b,c, we can see that the cell values around 5.0 m are large, while such noise components around 8.0 m scarcely appear. In particular, Figure 6c shows no noise component around 8.0 m at all, which is brought by noise reduction with DIP.

**Figure 7:**
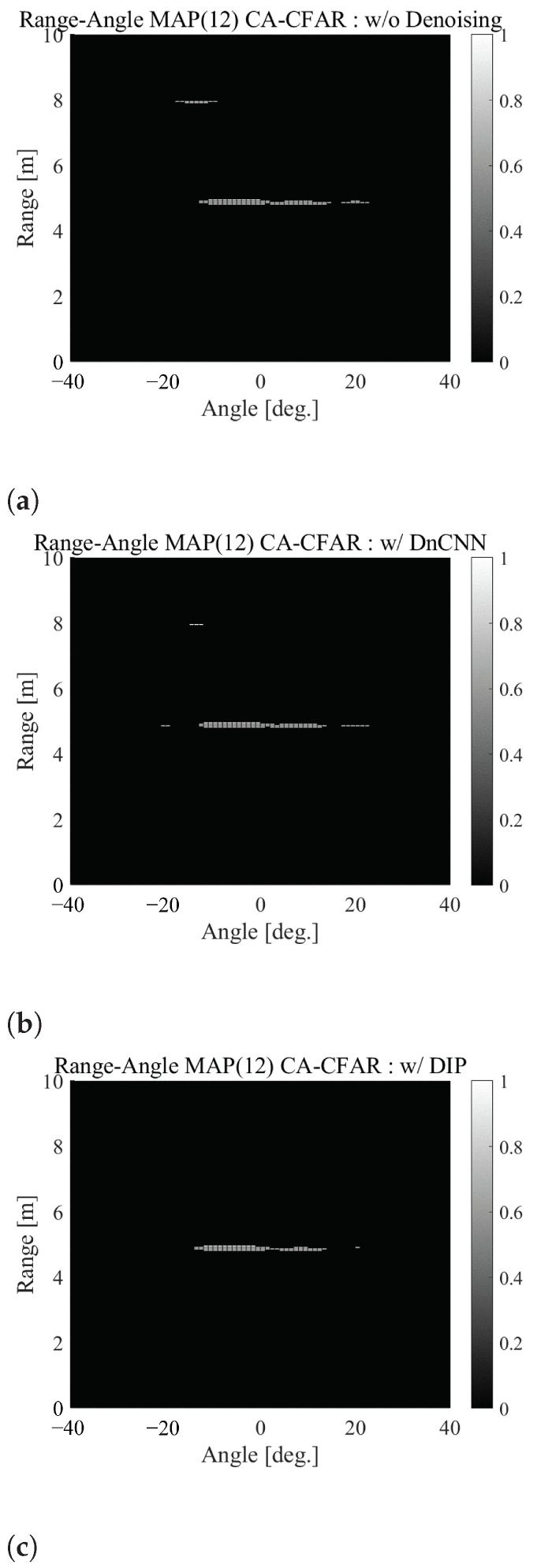
The comparison of detection maps (**a**) without denoising, (**b**) with DnCNN and (**c**) with DIP, where “1” means the detected cell, and “0” means the cell that is not detected.

Figure 7a–c shows the detection maps detected by CA-CFAR corresponding to Figure 6a–c, respectively. To generate these detection maps, we set the specified factor α to 1.6. As aforementioned, in the detection maps, “1” means the detected cell, and “0” means the cell that is not detected. As can be seen from these figures, the location where no one exists is detected around 8.0 m in Figure 7a. At the same location, false alarms are reduced in Figure 7b,c—particularly in Figure 7c, where they rarely occur. These results indicate that applying denoising, particularly DIP, to the range-angle map can reduce the effects of noise that could produce a false alarm.

#### 4.2.2. Detection Performance

Next, to evaluate the detection performance improved by denoising, we show the relation between the detection rate and false-alarm rate with and without denoising in the above case. Here, we define “detection” and “false alarm” as follows:

- If there are “1”s around the vicinity of each target in the detection map, the map is “detection”.
- If there is a “1” in other areas, the map is “false alarm”.

We calculated the detection rate $P_{d}$ and the false-alarm rate $P_{\mathrm{fa}}$ using 30 range-angle maps with and without denoising when α was varied from 1.0 to 9.9 by 0.1 steps as follows:

(8) $$\begin{array}{ccc} P_{d} & = & \frac{N_{d}}{N_{m}}, \end{array}$$

(9) $$\begin{array}{ccc} P_{\mathrm{fa}} & = & \frac{N_{\mathrm{fa}}}{N_{m}}, \end{array}$$

where $N_{d}$ is the number of range-angle maps determined as “detection”, $N_{\mathrm{fa}}$ is the number of range-angle maps determined as “false alarm” and $N_{m}$ is the total number of range-angle maps, i.e., 30. In all measurements, there were two people as targets. Therefore, in calculating $N_{d}$, one range-angle map was calculated as 1 if both targets were detected, 0.5 if only one target was detected and 0 if neither was detected, and the sum of these values was taken as $N_{d}$.

**Figure 8:**
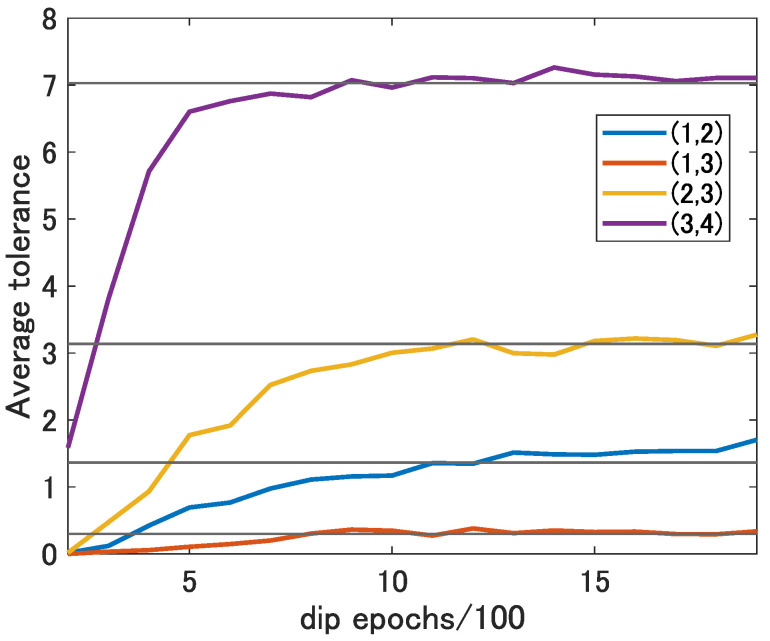
The average tolerance for each DIP epoch at each location.

Since the denoising effect of DIP differs depending on the epoch of DIP for each original image, it is necessary to select the best epoch for each original image to maximize the denoising effect. Figure 8 shows the average tolerance defined in Section 3.3 for each epoch of DIP in range-angle maps at each target location, which becomes wider as the number of epochs of DIP increases and which converges at a certain epochs. As the learning curve of the DIP converges as the epoch progresses as shown in [26], the tolerance converges as the epoch progresses.

The black lines in this figure show the tolerance of CFAR threshold parameter α without denoising at each location. Comparing the colored lines and black lines in each location, the average tolerance with DIP at about 1500 epochs exceeds those without denoising. Therefore, applying DIP for about 1500 epochs in the range-angle maps at each location is expected to give better performance than without denoising. Here, to check the effect of DIP, we manually selected the epoch that removed the most noise for each range-angle map and calculated the detection rate and the false-alarm rate using the denoised range-angle map.

**Figure 9:**
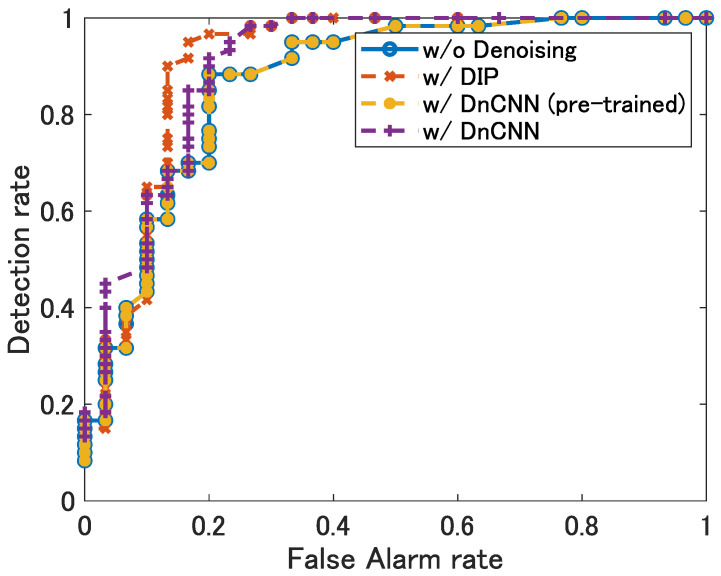
The ROC curves of the detection results by CA-CFAR without and with denoising at location (1,2).

Figure 9 shows the ROC curves, where the blue line shows the curve without denoising, the yellow line shows the curve with DnCNN using pre-trained network, the purple line shows the curve with DnCNN using the network trained by the range-angle map obtained by the experiments, and the red line shows the curve with DIP, respectively. The Area Under Curve (AUC) in this figure was 0.87 without denoising, 0.90 with DnCNN and 0.91 with DIP.

Table 3 shows the AUC for each target location. In the locations (1,2), (1,3) and (2,3), the AUCs were improved by DnCNN and DIP. In location (3,4), because the targets were at a close distance from the radar, they were always detected, resulting in an AUC of 1.00 with or without denoising. In location (2,4), the AUC was always 0.5 because the detection rate was always 0.5 regardless of the false-alarm rate. This may be due to the fact that the rear target location 4 was always undetected because it was hidden behind the front target location 2.

In location (1,4), the effect of the reflection from the wall was so strong that it could not be eliminated even by every denoising method, resulting in a significant degradation of the AUC. Here, Table 4 shows the AUC when the processed range of the range-angle map is set before 8.0 m. As can be seen from this table, it can be confirmed that, if the positions of reflective objects, such as a wall, are obtained as prior information and removed from the range-angle map in advance, the AUC is improved by denoising even at those positions (1,4). These results confirm that the detection performance was improved by denoising—particularly DIP.

#### 4.2.3. Tolerance of the CFAR Parameter Settings

Furthermore, to show the ease of threshold setting by the proposed method, we evaluated the performance for different α values used for the threshold setting of CA-CFAR. We referenced Figure 6 and Figure 7. As described in Section 3.3, if α is made smaller to ensure reliable target detection, the threshold value $V_{th}$ also becomes smaller, thereby, resulting in false detection due to noise. As a result, the α values that do not cause false alarms around 8.0 m are more than 2.1, 1.7 and 1.3 without denoising, with DnCNN and with DIP, respectively.

If α is increased to reduce false alarms due to noise, the threshold $V_{th}$ will also increase, making target detection impossible. Furthermore, α values to detect the two targets around 5.0 m are less than 3.1, 3.7 and 2.9 without denoising, with DnCNN and with DIP, respectively. In summary, the range of α where the targets can be detected without the false alarm is from 1.7 to 3.7 with DnCNN and from 1.3 to 2.9 with DIP, which is wider than 2.1 to 3.1 without denoising. This indicates that denoising can make the CA-CFAR threshold setting easy.

**Figure 10:**
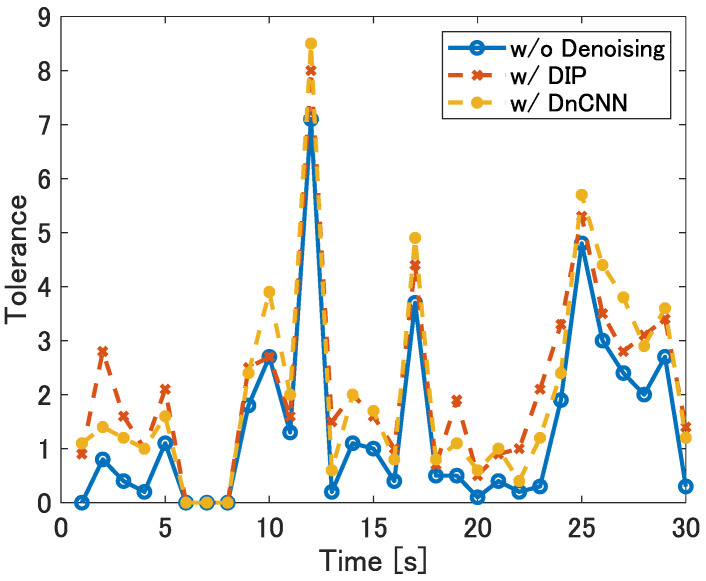
The tolerance of CA-CFAR parameter α with and without denoising at location (1,2).

To evaluate the ease of the CFAR threshold setting, we use “tolerance” defined as the width of the range of α within which targets are detected and false alarms are not generated in Section 3.3. In the results above, the tolerance is 1.0 when denoising is not applied, while the tolerance with DnCNN is 2.0, and the tolerance with DIP is 1.6. The tolerance varies with the range-angle map generated at every second. Figure 10 shows the tolerance at location (1,2) for each range-angle map—in other words, for each second. This result shows that DIP and DnCNN widens the tolerance of the individual range-angle maps.

**Figure 11:**
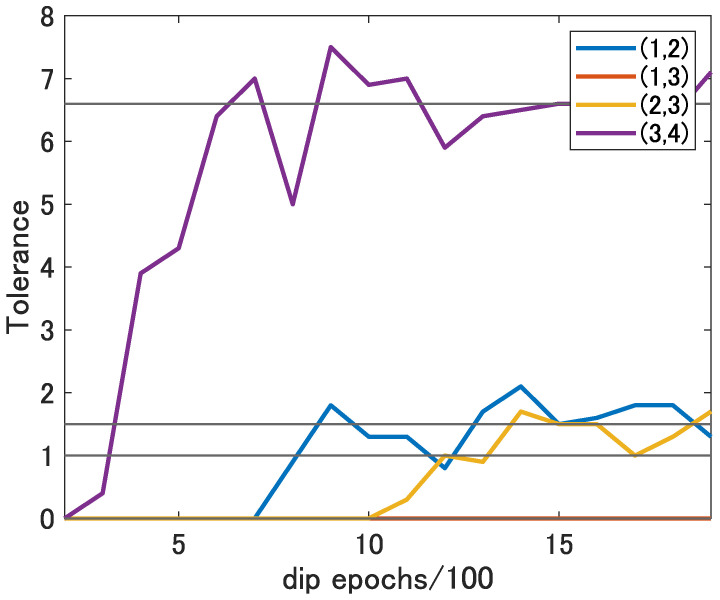
The tolerance in a range-angle map for each DIP epoch at each location.

Figure 11 shows the tolerance in a range-angle map at 15 s for each DIP epoch at each location. As in Figure 8, the black lines in this figure show the tolerance without denoising at each location. From this figure, it can be confirmed that the tolerance is better at 1400 epochs for DIP at locations (1,2) and (2,3) and at 900 epochs for DIP at location (3,4) than without denoising. Thus, by appropriately selecting the DIP epoch for each range-angle map, the tolerance can be improved. Table 5 shows the results of the average tolerance of the range-angle maps for all time at each target location. These results confirm that denoising makes the tolerance wider and that the tolerance of the α setting can be wider.

#### 4.2.4. SNR Analysis of the Range-Angle Maps

**Figure 12:**
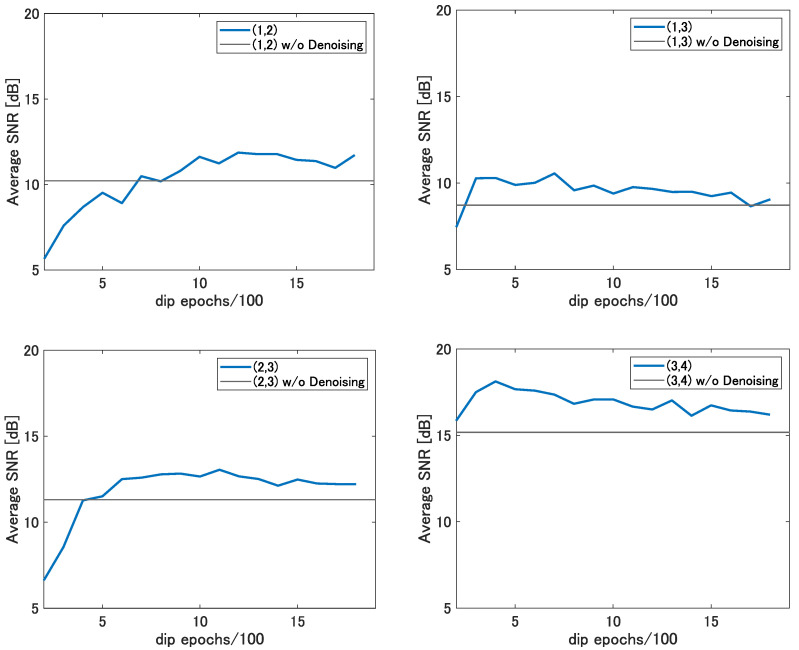
The average SNR for each DIP epoch at each location.

We evaluated the SNR of the range-angle maps before and after denoising was applied. The SNR was calculated as follows:

(10) $$\begin{array}{c} \mathrm{SNR}=20\log_{10}\frac{S}{N}\;\left[\mathrm{dB}\right], \end{array}$$

where *S* is the largest CL value in the area where the targets are located, and *N* is the largest CL value in the area other than the targets. Figure 12 shows the average SNR over the 30 range-angle maps at each target location. The blue lines show the average SNR for each epoch of DIP in range-angle maps, and the black lines show the average SNR without denoising. This figure shows that the application of DIP improves the SNR of the range-angle maps.

**Figure 13:**
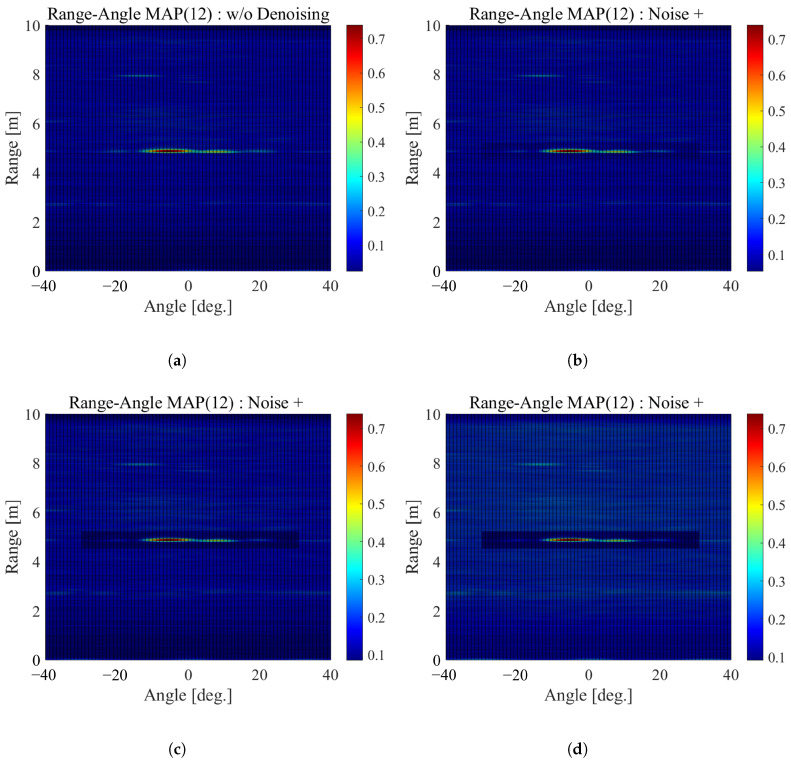

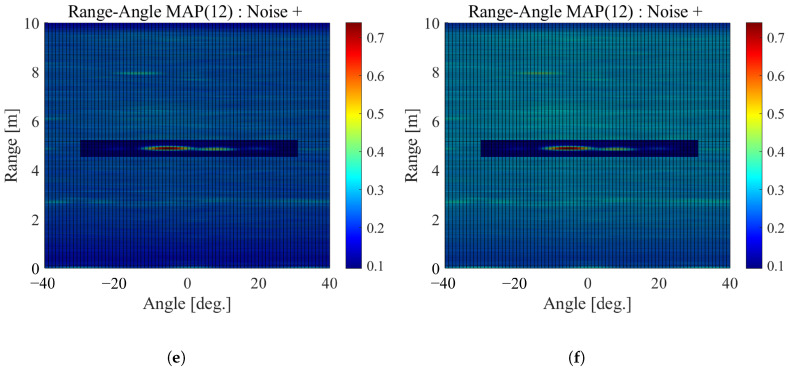
The range-angle maps with and without adding noise. (**a**) Without adding noise. (**b**) With adding 1 dB noise. (**c**) With adding 2 dB noise. (**d**) With adding 3 dB noise. (**e**) With adding 4 dB noise. (**f**) With adding 5 dB noise.

Next, we evaluated the effect of denoising by adding noise to the range-angle maps and varying the SNR. Here, we used the range-angle maps for location (1,2) with the farthest target location and the lowest SNR. Noise of 1 to 5 dB was added only to non-target areas. In other words, the SNR was intentionally degraded by 1 to 5 dB. Figure 13 shows examples of the range-angle maps with and without adding noise. Table 6 shows the AUC for adding noise at the target location (1,2).

The table shows that the AUC improved as the amount of noise added increased. As can be seen in the figure, this is because noise that could have been false alarms in areas other than the targets was buried by the added noise and was removed by CFAR because the CFAR threshold became high. Note that it was difficult to locate the target from the range-angle map in the low SNR environment when CFAR was not used. Although target-detection performance was improved despite the SNR degradation, denoising further improved the AUC in these environments.

## 5. Conclusions

In this paper, we proposed a method that improved the CFAR threshold tolerance by denoising a range-angle map using DIP for MIMO FMCW radar-signal processing. The proposed method applied DIP to a range-angle map generated using the CL method to reduce the effects of noise that could produce a false alarm. As a result of the noise reduction from the range-angle map by applying DIP, we showed that the target-detection performance—the AUC—was improved.

In addition, we confirmed that applying DIP improved the SNR of the range-angle map. However, false alarms still occur in extremely strong clutter environments, and detection is difficult when the target is hidden behind an object and when the signal is extremely weak. To detect targets in such an environment and to avoid false alarms, a method that emphasizes signals based on the characteristics of reflected signals from the human body and reduces other signals is necessary.

It is a future task to confirm to what extent the target-detection performance improves when such methods are combined with the proposed method. Through experiments to detect human locations in indoor environments, we showed that applying DIP to a range-angle map could reduce the number of false alarms, improve the detection performance and improve the tolerance of the CA-CFAR threshold setting.

## Figures and Tables

**Table 1:** Radar specifications.

Item	Value
Center frequency	79 GHz
Modulation method	FMCW
Bandwidth	3.4391 GHz
Sampling rate	160 Hz
Number of transmitting elements	3
Number of receiving elements	4
Number of virtual elements	12

**Table 2:** CA-CFAR parameters.

Item	Value
Guard Band cells (Range)	3
Guard Band cells (Angle)	7
Training Band cells (Range)	5
Training Band cells (Angle)	7

**Table 3:** AUC for each target location.

Locations	No Denoising	DIP	DnCNN (Pre-Trained)	DnCNN
(1,2)	0.87	0.91	0.87	0.90
(1,3)	0.70	0.76	0.70	0.71
(1,4)	0.05	0.05	0.02	0.00
(2,3)	0.97	0.99	0.97	0.98
(2,4)	0.50	0.50	0.50	0.50
(3,4)	1.00	1.00	1.00	1.00

**Table 4:** AUC for each target location (without the effect of the wall).

Locations	No Denoising	DIP	DnCNN (Pre-Trained)	DnCNN
(1,2)	0.96	0.98	0.96	0.99
(1,3)	0.77	0.85	0.77	0.80
(1,4)	0.78	0.87	0.77	0.55
(2,3)	0.98	1.00	0.99	0.99
(2,4)	0.50	0.50	0.50	0.50
(3,4)	1.00	1.00	1.00	1.00

**Table 5:** The average tolerance of CFAR parameter α.

Locations	No Denoising	DIP	DnCNN
(1,2)	1.4	2.1	2.0
(1,3)	0.3	0.7	0.3
(1,4)	0.0	0.1	0.0
(2,3)	3.1	4.1	4.0
(2,4)	0.0	0.0	0.0
(3,4)	7.0	7.8	6.6

**Table 6:** AUC for adding noise.

Adding Noise [dB]	No Denoising	DIP	DnCNN
0	0.87	0.91	0.90
1	0.94	0.96	0.95
2	0.97	0.98	0.99
3	0.98	0.99	0.99
4	0.99	0.99	1.00
5	0.99	0.99	0.98

## Data Availability

Not applicable.

## Abbreviations

The following abbreviations are used in this manuscript:

MIMO	Multiple-Input Multiple-Output
FMCW	Frequency-Modulated Continuous Wave
DOA	Direction of Arrival
SNR	Signal-to-Noise Ratio
CFAR	Constant False Alarm Rate
CL	Curve-Length
DIP	Deep Image Prior
DnCNN	Denoising Convolutional Neural Network
ROC	Receiver Operating Characteristic
CA-CFAR	Cell-Averaging Constant False Alarm Rate
AUC	Area Under Curve
